# Being Moved by Unfamiliar Sad Music Is Associated with High Empathy

**DOI:** 10.3389/fpsyg.2016.01176

**Published:** 2016-09-15

**Authors:** Tuomas Eerola, Jonna K. Vuoskoski, Hannu Kautiainen

**Affiliations:** ^1^Department of Music, Durham UniversityDurham, UK; ^2^Department of Music, University of JyväskyläJyväskylä, Finland; ^3^Faculty of Music, University of OxfordOxford, UK; ^4^Department of General Practice and Primary Health Care, University of HelsinkiHelsinki, Finland

**Keywords:** music, emotion, sadness, empathy, felt experience, individual differences, being moved

## Abstract

The paradox of enjoying listening to music that evokes sadness is yet to be fully understood. Unlike prior studies that have explored potential explanations related to lyrics, memories, and mood regulation, we investigated the types of emotions induced by unfamiliar, instrumental sad music, and whether these responses are consistently associated with certain individual difference variables. One hundred and two participants were drawn from a representative sample to minimize self-selection bias. The results suggest that the emotional responses induced by unfamiliar sad music could be characterized in terms of three underlying factors: Relaxing sadness, Moving sadness, and Nervous sadness. Relaxing sadness was characterized by felt and perceived peacefulness and positive valence. Moving sadness captured an intense experience that involved feelings of sadness and being moved. Nervous sadness was associated with felt anxiety, perceived scariness and negative valence. These interpretations were supported by indirect measures of felt emotion. Experiences of Moving sadness were strongly associated with high trait empathy and emotional contagion, but not with other previously suggested traits such as absorption or nostalgia-proneness. Relaxing sadness and Nervous sadness were not significantly predicted by any of the individual difference variables. The findings are interpreted within a theoretical framework of embodied emotions.

## 1. Introduction

The human ability to derive genuine pleasure from tragedy and sadness portrayed in fiction has been acknowledged for millennia (e.g., the writings of Aristotle, Nietzsche, Schopenhauer, and Hobbes). The paradoxical enjoyment of negative emotions such as sadness has attracted contemporary attention in diverse fields such as media psychology (Schramm and Wirth, 2010), philosophy (Knobloch-Westerwick et al., 2013), psychology (Wildschut et al., 2006; Goldstein, 2009), and neuroscience (Wagner et al., 2014). However, the underlying mechanisms for this paradox still remain unclear. Listening to sad music is one particularly striking example of this phenomenon; it is not only common for listeners to report feelings of sadness induced by sad music (Juslin et al., 2011), but these experiences are typically described to be highly enjoyable (Eerola and Peltola, 2016; Peltola and Eerola, 2016). Furthermore, most cultures have distinct types of sad music (Agawu, 1988) that can be recognized from relatively simple acoustic cues even without appropriate cultural knowledge (Laukka et al., 2013).

The reasons for listening to self-identified sad music have been explained in terms of mood regulation (Garrido and Schubert, 2011b; Van den Tol and Edwards, 2015), mood congruency (Hunter et al., 2011; Taruffi and Koelsch, 2014), and autobiographical memories (Vuoskoski and Eerola, 2012; Tahlier et al., 2013), which suggest that the appeal is external to music. In these explanations, music is typically familiar to listeners, and considered to be either a vehicle to reminisce (Tahlier et al., 2013), to reflect on ideas conveyed by the lyrics (Mori and Iwanaga, 2014), or to derive comfort from Van den Tol and Edwards (2013). All these mechanisms seem to be unrelated to music itself, suggesting that any (familiar) music may be able to deliver the desired effects (Juslin and Västfjäll, 2008). However, anecdotes and descriptions of strong experiences with music (SEM) by Gabrielsson (2011) suggest that unfamiliar music might also lead to tears and strong, positive emotions without direct reference to any external sources. Oliver Sacks offers a compelling example from his own life; after the death of a close relative, the ensuing anhedonia came to an end one evening when “the whole concert bored me—until the last piece was played. It was a piece I had never heard before, by a composer I had never heard of […]. Suddenly, as I listened, I found my eyes wet with tears” (Sacks, 2007, p. 296–297).

How could such experiences be explained if they cannot be attributed to any external sources? Juslin (2013) has proposed that aesthetic appreciation might play a role, and that the “paradoxical” enjoyment of music-induced sadness has in fact nothing to do with sadness—the percept of sadness only happens to co-occur with the percept of beauty. A related account is given by Hanich et al. (2014), who demonstrated that enjoyment derived from sad films is mediated by feelings of being moved, not directly by sadness itself. The few empirical studies that have explored the appeal of unfamiliar sad music (Vuoskoski and Eerola, 2011; Vuoskoski et al., 2012) have indeed discovered that there appears to be an association between aesthetic appreciation and perceived sad emotional expression in music. However, these studies have also revealed that there is significant inter-individual variability in the enjoyment of sad music, and that these individual differences—related to both enjoyment and the intensity of emotional responses—are associated with certain personality traits—most notably trait empathy (Garrido and Schubert, 2011a; Vuoskoski et al., 2012). Trait empathy relates to an individual's dispositional responsiveness to the observed (negative) experiences of others, and involves both emotional and cognitive components (Eisenberg et al., 1994). This connection between empathic responsiveness and the enjoyment of sad music suggests that there is something about the emotional qualities of sad music—not just its aesthetic qualities—that appeals to certain listeners and engages them.

The principal mechanisms through which unfamiliar sad music evokes sadness in listeners are thought to involve empathy and emotional contagion (Juslin and Västfjäll, 2008; Vuoskoski and Eerola, 2012). Both empathy and emotional contagion lead to the experience of the same emotion as perceived in the object of attention, but only empathy involves the conscious awareness of the perceived emotional expression being the source of one's own emotional response. Emotional contagion is thought to be a pre-conscious process that involves mimicry and embodied simulation of perceived emotional cues (Hatfield et al., 1994). Trait empathy has previously been associated with reflexive mimicry of emotional facial expressions (Sonnby-Borgström et al., 2003), and stronger somatomotor responses to others' pain (Avenanti et al., 2009). Thus, it is plausible that listeners with high trait empathy also engage in embodied simulation of sad expressive cues in the context of music listening, potentially impacting the type and intensity of emotions experienced.

The aim of the present study is to explore the range of experiences evoked by listening to nominally sad, unfamiliar music, and to identify the individual difference variables that reliably predict the enjoyment of music-induced sadness. By focusing on unfamiliar, instrumental music, we can eliminate most external sources of emotions (such as episodic memories, lyrics, and contextual associations).

## 2. Materials and methods

### 2.1. Ethics statement

All participants gave their written, informed consent in compliance with an experimental protocol approved by the Ethics Committee of University of Jyväskylä, Finland. The methods were carried out in accordance with the approved guidelines.

### 2.2. Participants

A representative sample (*N* = 1500) of people living in Finland was obtained via Statistics Finland. The sample was stratified according to region, age (18–67 years), and gender. From this pool, volunteers were recruited in major cities. This yielded a total of 102 participants, out of which 65 were women. The age of the participants ranged from 20 to 67 (*M* = 43.4, *SD* = 14.7). All participants received a monetary compensation (15 EUR) in return for their participation.

### 2.3. Musical stimulus

An eight and a half-minute excerpt from the instrumental piece *Discovery of the Camp* (Band of Brothers soundtrack by Kamen, 2001) was used as the unfamiliar sadness-evoking stimulus. This excerpt has been previously validated, and successfully used to induce sadness in participants (Vuoskoski and Eerola, 2012, 2015).

### 2.4. Measures

#### 2.4.1. Self-reports

Participants current mood (both before and after music listening) was measured using the Positive and Negative Affect Schedule (PANAS, Watson et al., 1988). Participants also provided ratings regarding the emotions that they perceived and felt during the music listening task. First, participants were asked to rate whether or not the music evoked an emotional response in them, and how strong that response was (i.e., intensity of felt emotion). Next, they were asked to describe their felt and perceived emotions using adjective slider scales. The difference between felt and perceived emotions was made clear to them (i.e., “How did you feel when you listened to the music?” vs. “How did the music sound like?”). The scale labels for felt emotions were peaceful, anxious, moved, and sad, (the extremes of the scales were labeled “Does not describe my emotional reaction at all” and “Describes my emotional reaction very well”), while the scale labels for perceived emotions were peaceful, positive, negative, scary, and sad (the scale extremes were “Does not describe the music at all” and “Describes the music very well”). The scales provided output in the range of 0–127, but the data has been rescaled for this manuscript so that 0 reflects the low extreme and 10 the high extreme. In addition, participants were asked to rate how much they liked the excerpt. The selection of rating scales was based on past studies (Vuoskoski and Eerola, 2011; Vuoskoski et al., 2012).

#### 2.4.2. Indirect measures

To complement the self-reports of felt emotion, a pictoral facial expression judgment task was used to measure participants' emotional states in a more implicit, indirect manner. Participants were presented with 25 composite pictures depicting prototypical and ambiguous facial expressions (Vanger et al., 1998), and asked to rate the emotions expressed by the facial pictures using five slider scales (tender, sad, scary, angry, and neutral). According to the affect-congruency theory (Bower, 1981), people experiencing a sad affective state should perceive more sadness in emotional stimuli, and thus exhibit a judgment bias toward sadness in emotional judgment tasks. An identical task has previously been used in the context of sad music listening (Vuoskoski and Eerola, 2012, 2015). To eliminate the effect of possible individual differences in scale use, the raw emotion ratings for the facial expression pictures were standardized within subjects using individual z-score transformations. The z-scores were calculated using all emotion ratings (ratings of sadness, happiness, anger, fear, and neutrality perceived in the facial pictures) of each participant.

#### 2.4.3. Psychophysiological measures

Two psychophysiological indices of experienced emotion were recorded during the music listening task. Electrodermal activity (EDA) is an indicator of sympathetic activity, and has been shown to reflect emotional responses induced by musical stimuli, including sad music (Gomez and Danuser, 2004; Baumgartner et al., 2006; Juslin et al., 2013). EDA was measured from the distal phalanges of the index and middle fingers. Heart rate variability (HRV) is the temporal variation in the intervals between consecutive heartbeats. Heart is modulated by the autonomic nervous system (ANS), and HRV is assumed to reflect both parasympathetic and sympathetic substrates of the ANS (Quintana and Heathers, 2014). The most powerful analysis techniques of HRV rely on spectral analysis of particular frequency bands, best extracted with wavelet transforms. HRV was measured using two electrodes placed at the upper (chest) and lower torso (below the heart), recording the electrocardiogram (ECG) signal.

EDA and ECG signals were recorded using a portable NeXus-10 MKII system with BioTrace+ software (*Mind Media BV*, Netherlands). For both measures, the sampling rate was 256 samples per second. The ECG data was subjected to high (0.002 Hz) and low-pass filters (10 Hz) before the actual HRV analysis. Since the role of the physiological measures was ancillary to self-reported emotions, they were only collected from a subsample of 44 participants. This sample was estimated to provide a statistical power of 0.90 for detecting an effect size of 0.50 in a within-participants design (Juslin et al., 2013). Even though the experiment was run in four different major cities, physiological indices were only collected in the first two cities.

Four components of EDA were extracted using the Matlab-toolbox Ledalab (Benedek and Kaernbach, 2010). The tonic and phasic components of EDA were decoupled by means of continuous deconvolution analysis, yielding three measures related to phasic skin conductance responses (the number, mean height, and speed of SCR peaks), and one measure reflecting the tonic component of EDA, (median skin conductance level). All four components are positively related to sympathetic activity, and should thus decrease with relaxation and increase with activation and stress. Four spectral components of HRV were extracted; Low frequency (LF) component (0.04–0.15 Hz), high frequency (HF) component (0.15–0.40 Hz), very low frequency (VLF) component (< 0.03 Hz), and low frequency component to high frequency ratio (LF/HF), estimated with wavelet transforms (Mendez et al., 2014). High values in VLF, LF, and LF/HF are associated with the activity of the sympathetic nervous system, whereas HF is associated with the parasympathetic nervous system, although the specifics and interactions between the components have been debated (Akselrod et al., 1981). Both sets of indices were extracted from epochs of equal duration (2 min and 30 s), each epoch starting 10 and 60 s after the beginning of the Condition (Baseline or the Music, respectively).

#### 2.4.4. Measures of individual differences

To explore the role of individual differences in enjoyment music-induced sadness, a number of previously implicated personality variables were collected. Since empathy has been suggested to play a role in the enjoyment of sad music (Garrido and Schubert, 2011a; Vuoskoski and Eerola, 2011), a widely used measure of trait empathy (*Interpersonal Reactivity Index*, IRI, Davis, 1983) as well as measures of related traits such as *Emotional Contagion* (ECS, Doherty, 1997) and *Absorption* (ABS, Glisky and Kihlstrom, 1993), were administered. Since proneness to nostalgia has also been linked with music and sadness (Barrett et al., 2010), the *Southampton Nostalgia Scale* (SNS, Routledge et al., 2008) was also administered to all participants. The participants filled in a subjective evaluation of their health using the *General Health Questionnaire* (Goldberg and Williams, 1988), since it is known to be negatively associated with perception, appraisal, and control of emotions (Tsaousis and Nikolaou, 2005). Finally, several music-related measures were administered: Music preferences was measured using a simple listing of liked and disliked artists (Ferrer et al., 2013), since preference is known to be associated with emotional intensity of unfamiliar music (Ladinig and Schellenberg, 2012). A *Measure of Musical Sophistication* (Ollen, 2006) indexed the expertise in music, sometimes shown to be related to emotional responses to music (Ladinig and Schellenberg, 2012). Two instruments directly aimed to explore music and sadness were collected to be comparable with past studies; the *Attitudes toward Sad Music* (ASM, Eerola et al., 2015) has six attitude dimensions that relate to music and the *Like Sad Music Scale* (LSMS, Garrido and Schubert, 2013) has nine questions about preferences for sad music. Finally, information about the demographic details of the participants—their age, gender, and education—was also collected. All instruments were translated to Finnish.

### 2.5. Procedure

The experiment was conducted individually for each participant using a computer interface in a quiet room. After providing the participant with instructions and attaching the physiological sensors, the experiment started with a period of rest (3 min, a baseline for the physiological measures). After the baseline rest period, participants rated their current affective state using the 20 adjectives of the PANAS. Next, participants listened to the sad music excerpt, which lasted 8 min and 30 s. They were instructed to let themselves be immersed in the music. Participants listened to the music through studio-quality headphones, and they could adjust the volume according to their own preferences.

Directly after the music listening task, participants completed the indirect facial expression judgment task, followed by the self-ratings of current mood (PANAS), felt and perceived emotions, and liking. When the participants had completed the ratings, the physiological sensors were detached, and participants were moved to an office desk to complete the background questionnaires and to be debriefed.

## 3. Results

The background variables—general health (GHQ), trait empathy (IRI), absorption (ABS), emotional contagion (ECS), and nostalgia-proneness (SNS),—were screened for outliers and strong deviations from normality, but none were found. The inter-rater agreement was high for most instruments (see Table 1), and within the normal range for the measures. Also, the age distribution of the participants was similar to the national sample [$\chi_{\left(6\right)}^{2}$ = 12.4, *p* = ns], although the gender distribution deviated between the population and sample [$\chi_{\left(1\right)}^{2}$ = 8.33, *p* < 0.01] since more women volunteered for the experiment than men. The emotion and pre- and post-mood ratings (also displayed in Table 1) displayed high consistencies and expected pattern of means (Tahlier et al., 2013). The emotion ratings were combining into three constructs, explained in the next analysis, to estimate the internal consistency.

**Table 1:** **Instrument details (means, standard errors, and internal consistency)**.

	**Mean (SEM)**	**Internal consistency**
**BACKGROUND VARIABLES**		
GHQ	1^†^	0.85
IRI-Empathy	93.93 (1.04)	–
IRI-Fantasy	24.82 (0.48)	0.79
IRI-Perspective	24.21 (0.38)	0.70
IRI-Concern	26.54 (0.39)	0.77
IRI-Distress	18.36 (0.49)	0.82
ABS	44.21 (0.63)	0.78
ECS	53.46 (0.67)	0.77
SNS	20.43 (0.49)	0.90
**SELF-REPORTS OF EMOTIONS**		
Intensity (felt)	6.45 (0.22)	0.77^b^
Peaceful (felt)	6.44 (0.30)	0.89^a^
Anxious (felt)	0.64 (0.13)	0.82^c^
Moved (felt)	4.13 (0.33)	0.77^b^
Sad (felt)	3.98 (0.35)	0.77^b^
Peaceful (perceived)	6.05 (0.28)	0.89^a^
Positive (perceived)	4.64 (0.31)	0.89^a^
Negative (perceived)	1.02 (0.19)	0.82^c^
Scary (perceived)	1.24 (0.21)	0.82^c^
Sad (perceived)	6.03 (0.33)	0.77^b^
Liking	3.98 (0.35)	0.77^b^
**MOOD**		
Pre-positive	35.16 (0.63)	0.89
Post-positive	34.53 (0.71)	0.88
Pre-negative	13.52 (0.44)	0.91
Post-negative	12.88 (0.42)	0.89

a−c *consistency for emotions within Factors 1–3 from Table 2)*.

† *Median value due to the skewed distribution*.

### 3.1. Structure of emotional experiences

In order to explore the underlying structure of emotional responses evoked by the sad music excerpt, participants' ratings of perceived and felt emotion were subjected to factor analysis. The factorability of the rating scales was evaluated in terms of sampling adequacy. The Kaiser-Meyer-Olkin measure of sampling adequacy was 0.78, well above the recommended value of 0.60. Furthermore, outlier screening yielded no evidence of abnormalities. Given these indicators, factor analysis was conducted with all 11 scales. The number of factors was determined by parallel analysis (PA) algorithm (van der Eijk and Rose, 2015), which suggested three components using principal axis factoring method. To facilitate the interpretation of the factors and to acknowledge that emotion dimensions are typically non-orthogonal (Crawford and Henry, 2004), oblique rotation (oblimin) was applied. Three factors explained a total of 67% of the variance, and provided a generally good fit to the original data (RMSR = 0.04, χ^2^ = 79.5, *p* < 0.001). Since oblique rotation was used, we also explored the correlations between the factors; Factor 1 and 2, *r*_(99)_ = 0.23, *p* < 0.05; Factor 1 and 3, *r* = −0.63, *p* < 0.001; Factor 2 and 3, *r* = 0.08, *p* = ns. The analysis revealed a high negative correlation between factors 1 and 3.

**Figure 1:**
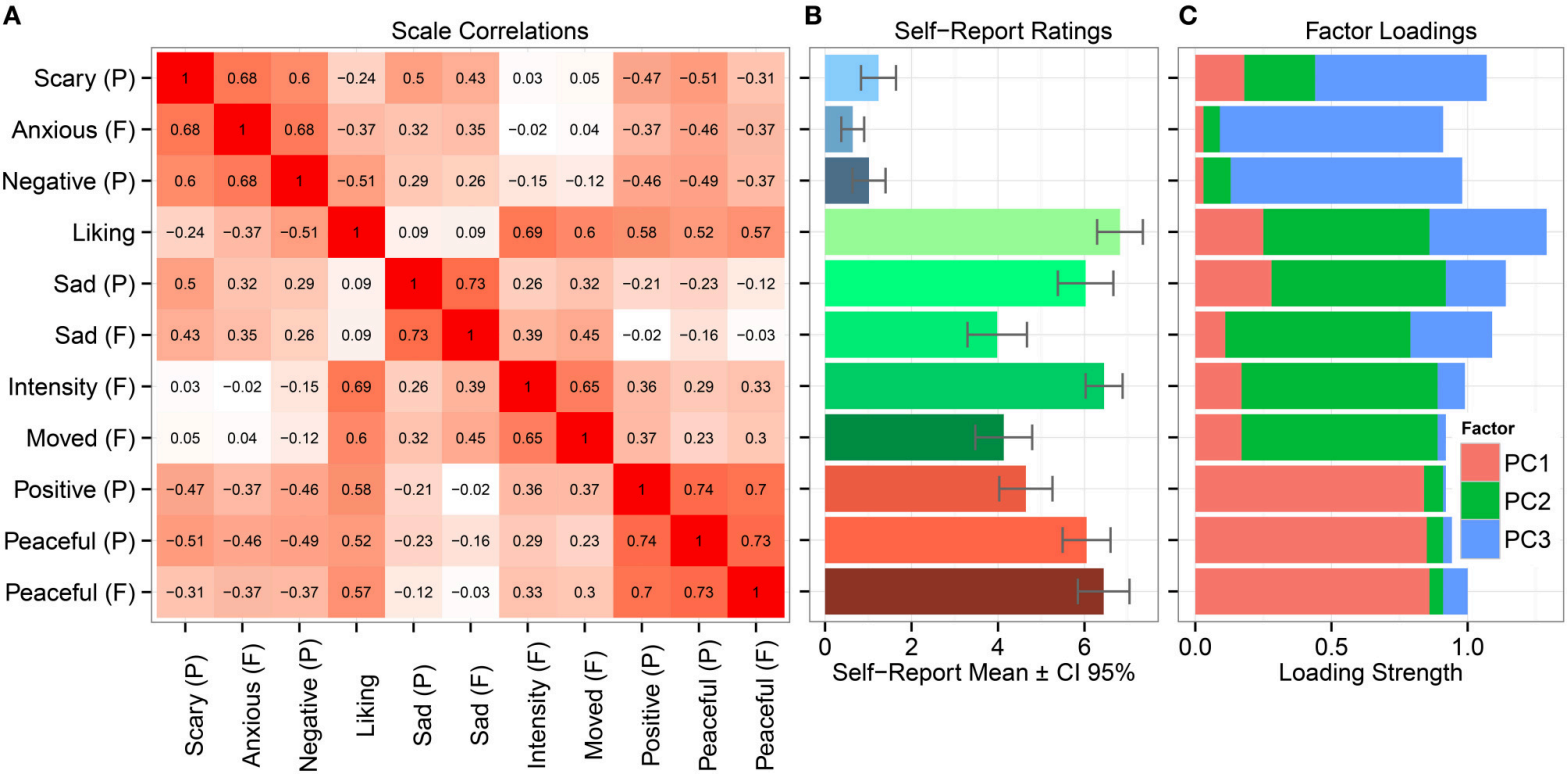
**(A)** Correlations between the self-report scales of emotions, **(B)** the mean ratings of self-report scales, and **(C)** the factor loadings of the scales.

The factor loadings are shown in Table 2. Factor 1, labeled as *Relaxing sadness*, is clearly related to positive, peaceful and relaxing emotional responses to sad music. Factor 2 obtains high loadings from the scales Moved (felt), Intensity (felt), Sad (felt and perceived), and Liking. This suggests a complex and intense emotional experience, involving both aesthetic, enjoyable emotions (such as liking and being moved; Hanich et al., 2014) and feelings of sadness. Thus, we labeled this factor as *Moving sadness*. Factor 3, received high loadings from scales related to negative, aversive experiences; Anxious (felt), Negative (perceived), and Scary (perceived) and was thus labeled as *Nervous sadness*. However, it should be noted that these scales received very low mean ratings (see Table 2), and thus the factor may have limited relevance in terms of participants' actual emotional responses. Conversely, Factors 1 and 2 seem to have to most relevance for the overall emotional experience evoked by the sad-sounding musical stimulus, since the scales loading to these two factors obtained the highest means ratings. Figure 1 summarizes the covariance pattern of the scales, the means, and the loadings in the factor solution.

**Table 2:** **Factor structure of self-reports of emotions (loadings < 0.35 not shown)**.

**Rating scale**	**Relaxing Sadness**	**Moving Sadness**	**Nervous Sadness**
Peaceful (felt)	0.86		
Peaceful (perceived)	0.85		
Positive (perceived)	0.84		
Moved (felt)		0.72	
Intensity (felt)		0.72	
Sad (felt)		0.68	
Sad (perceived)		0.64	
Liking		0.61	−0.43
Negative (perceived)			0.85
Anxious (felt)			0.82
Scary (perceived)			0.63
Loadings	2.60	2.42	2.31
Variance explained	24%	22%	21%

As previous research has suggested that emotional experiences induced by music differ across certain demographic variables (Garrido and Schubert, 2011a; Ladinig and Schellenberg, 2012), the factor scores were subjected to an ANOVA with Gender, Age (binned into 7-year categories), and Musical Expertise (5 levels) as the between-subjects factors. For factors 1 (*Relaxing sadness*) and 3 (*Nervous sadness*), no significant main effects emerged. For Factor 2 (*Moving sadness*), a main effect of Gender was observed, *F*_(1, 63)_ = 10.5, *p* = 0.002, $\eta_{G}^{2}$ = 0.14, women obtaining higher (*M* = 0.24, *SEM* = 0.12) scores than men (*M* = −0.43, *SEM* = 0.15).

### 3.2. Indirect measures

The mean standardized sadness ratings (*M* = −0.095, *SD* = 0.17) yielded by the facial expression judgment task (measuring felt sadness in an indirect manner) were comparable to those obtained in previous studies using similar musical stimuli (Vuoskoski and Eerola, 2012, 2015), not indicating a clear bias toward perceived sadness (relative to ratings of perceived happiness, anger, fear, and neutrality). However, as previous work has shown that there is significant inter-individual variability in the extent of this bias when listening to unfamiliar sad-sounding music (Vuoskoski and Eerola, 2012), we were especially interested in investigating whether the indirectly measured sadness correlated with self-reported emotions. Thus, Pearson correlation coefficients between the factor scores and mean sadness ratings (for facial pictures) were calculated. The degree of judgment bias toward sadness was positively associated with *Nervous sadness* (*r* = 0.22, *p* = 0.025), but not with *Moving sadness* (*r* = 0.19, *p* = 0.059) or *Relaxing sadness* (*r* = −0.08, *p* = 0.43). A similar analysis was carried out for mean happiness ratings, revealing a negative correlation with *Nervous sadness* (*r* = −0.26, *p* = 0.008), but no significant correlations with *Moving sadness* (*r* = −0.01, *p* = 0.90) or *Relaxing sadness* (*r* = 0.16, *p* = 0.11). After the Bonferroni method is used to adjust the threshold of statistical significance (to account for multiple tests; 0.05/6 = 0.008), only the negative correlation between *Nervous sadness* and perceived happiness remains statistically significant. These results indicate that those who reported experiencing more *Nervous sadness* also exhibited a more pronounced judgment bias toward sadness (as evidenced by the decreased evaluations of happiness in the facial pictures). The degrees of judgment biases toward sadness and happiness were not associated with mood prior to music listening (all *p*s > 0.20).

### 3.3. Physiological measures

In order to complement and corroborate the self-reports and indirect measures of experienced emotions, two emotion-related indices of ANS activity, electrodermal activity (EDA) and HRV, were analyzed. Felt emotions evident in the self-reports should also be—at least to a certain extent—reflected by electrophysiological indices when music listening is compared to silent baseline (Gomez and Danuser, 2004; Juslin et al., 2013). However, it is unclear whether responses to sad music would be more consistent with relaxation and relevant parasympathetic activity (Iwanaga et al., 2005), or whether sad music actually engages listeners and induces increased sympathetic activity (Gomez and Danuser, 2004).

Outliers were identified using the Grubbs method at *p* < 0.01 level, and cases violating the criteria (6.25% of the observations) were constrained to 2.5 SDs (Grubbs, 1969). Repeated-measures ANCOVAs with Condition (Baseline vs. Music) as a within-participants factor, Age, and Gender as between-participants factors, and factor scores of *Moving sadness* as a covariate, were conducted across all physiological measures. These analyses yielded main effects of Condition for VLF, *F*_(40, 1)_ = 12.0, *p* = 0.0013, $\eta_{G}^{2}$ = 0.073, and LF/HF *F*_(40, 1)_ = 7.25, *p* = 0.0103, $\eta_{G}^{2}$ = 0.056, but no interactions were evident. HF and LF components showed marginal, non-significant effects of Condition (and no other main or interaction effects). Both HRV measures were higher in the Music (VLF M = 264.97, SEM = 39.62, LF/HF M = 3.03, SEM = 0.35) than in the Baseline Condition (VLF M = 145.83, SEM = 24.65, LF/HF M = 2.12, SEM = 0.23), suggesting that the music listening did not evoke relaxation since it increased sympathetic nervous system activation.

For the four EDA components, similar ANOVA analyses with Condition (within-participants factor), Age, Gender (between-participants factors), and *Moving sadness* (covariate) yielded non-significant main and interaction effects (*p*s > 0.20).

To conclude, the self-reports of felt emotions suggesting that unfamiliar sad music was experienced as intense and highly moving were partly confirmed by spectral HRV components, which suggested that music listening lead to increased sympathetic nervous system activity. However, not all physiological indices supported this interpretation. For instance, EDA measures failed to differentiate music listening from the baseline condition.

In the subsequent analyses, we will explore the background variables associated with different emotional responses evoked by nominally sad music. Particular emphasis will be given to experiences of *Moving sadness*. This factor is characterized by intense experiences of being moved, and is thus consistent with the physiological indices demonstrating high sympathetic nervous in comparison to baseline. *Moving sadness* was also associated with higher liking ratings for the nominally sad excerpt, suggesting that this factor is in the crux of the paradox of how listeners are able to derive pleasure from sad experiences in musical contexts.

### 3.4. Identification of key variables associated with emotion factors

First, raw correlations between the factor scores of *Moving sadness* and background measures were calculated. Four variables correlated significantly with the self-reported experiences of *Moving sadness*; IRI-Fantasy (*r*_(99)_ = 0.38, *p* < 0.001), IRI-Concern (*r* = 0.35, *p* < 0.001), Emotional Contagion (*r* = 0.35), and positive mood prior to music listening (*r* = 0.21, *p* < 0.05). Also, the global measure of trait empathy (combination of the IRI subscales) correlated positively with *Moving sadness, r*_(99)_ = 0.38, *p* < 0.001.

A similar correlation analysis was carried out to explore the background variables associated with experiences of *Relaxing sadness*. This yielded positive correlations with *Quality of Life* [*r*_(99)_ = 0.24, *p* < 0.05] and positive mood prior to music listening [*r*_(99)_ = 0.25, *p* < 0.05], and a negative correlation with negative mood [*r*_(99)_ = −0.20, *p* < 0.05].

Correlations between *Nervous sadness* and background variables displayed a negative association with *Quality of Life* [*r*_(99)_ = −0.24, *p* = 0.016], and a positive correlation between the factor scores and *Negative mood* [*r*_(99)_ = 0.27, *p* < 0.01].

Since the experience of *Moving sadness* varied notably across the participants, and as past studies have suggested that music-induced sadness is only found pleasurable by some listeners (Vuoskoski and Eerola, 2012; Eerola et al., 2015), we set out to assess the discriminatory power of the key background variables in predicting *Moving sadness*. First, we identified those who are particularly strongly moved by sad music, and experience intense yet sad emotions—hereafter labeled as “sadness enjoyers”—by selecting the participants whose factor scores for *Moving sadness* were in the top quintile (20 of 101 participants). Membership in the sadness enjoyers group was then predicted with each of the four background variables that correlated (*r* > |0.30|) significantly with *Moving sadness*. This analysis will first be carried across all participants, and then separately for men and women, since gender was found to have a significant impact on the factor scores of *Moving sadness*.

The prediction of group membership (“sadness enjoyers”) was carried out using receiver operating characteristic (ROC) curves that offer a robust method for guarding against false attributions (false negatives and positives) and provide optimal cutpoints and diagnostics for the predictors (Pepe, 2003). Table 3 displays the results of this analysis for each of the four predictors. As the AUC values (i.e., area under the curve; the probability that a classifier will rank a positive instance higher than a negative one) Likelihood Ratios suggest, most of the variables were able to discriminate sadness enjoyers from the rest of the participants. Overall, global empathy (*IRI-Empathy*, AUC = 72.3, CI_95_ = 59.7–84.9) and *IRI-Fantasy* subscale (AUC = 71.0, CI_95_ = 56.9–85.1) were the best predictors.

**Table 3:** **Classification rates and cut-off values of the sadness enjoyers with the key background variables**.

**Variable**	**AUC (CI_95_)**	**Cut-off**	**Sensitivity (CI_95_)**	**Specificity (CI_95_)**	**LR**
*IRI-Empathy*	72.3 (59.7–84.9)	100	65.2 (40.8–84.6)	77.8 (67.2–86.3)	2.93
*IRI-Fantasy*	71.0 (56.9–85.1)	27	55.5 (31.5–76.9)	84.0 (74.1–91.2)	3.43
*IRI-Concern*	65.9 (51.5–80.4)	28	35.6 (15.4–59.2)	92.6 (84.6–97.2)	4.73
*ECS*	67.2 (54.7–79.7)	56	60.1 (36.1–80.9)	69.1 (57.9–78.9)	1.94

*AUC, Area Under Curve; LR, Positive Likelihood Ratio*.

In general, the classification accuracy of sadness enjoyers is lower for men (AUCs of 48.0, 40.4, 52.5, and 45.1 for *IRI-Empathy, IRI-Fantasy, IRI-Concern*, and *ECS*, respectively). The lower prediction rate of men enjoying music-induced sadness may be related to the number of men in the sample (36.3%), to the lower thresholds that the variables had for men, or the higher range of the factor scores for women. Women typically obtain higher scores of self-reported empathy than men (Tobin et al., 2000; Derntl et al., 2010), but whether these differences also exist on the level of behavior (and in the enjoyment of empathy-related activities) is unclear.

**Figure 2:**
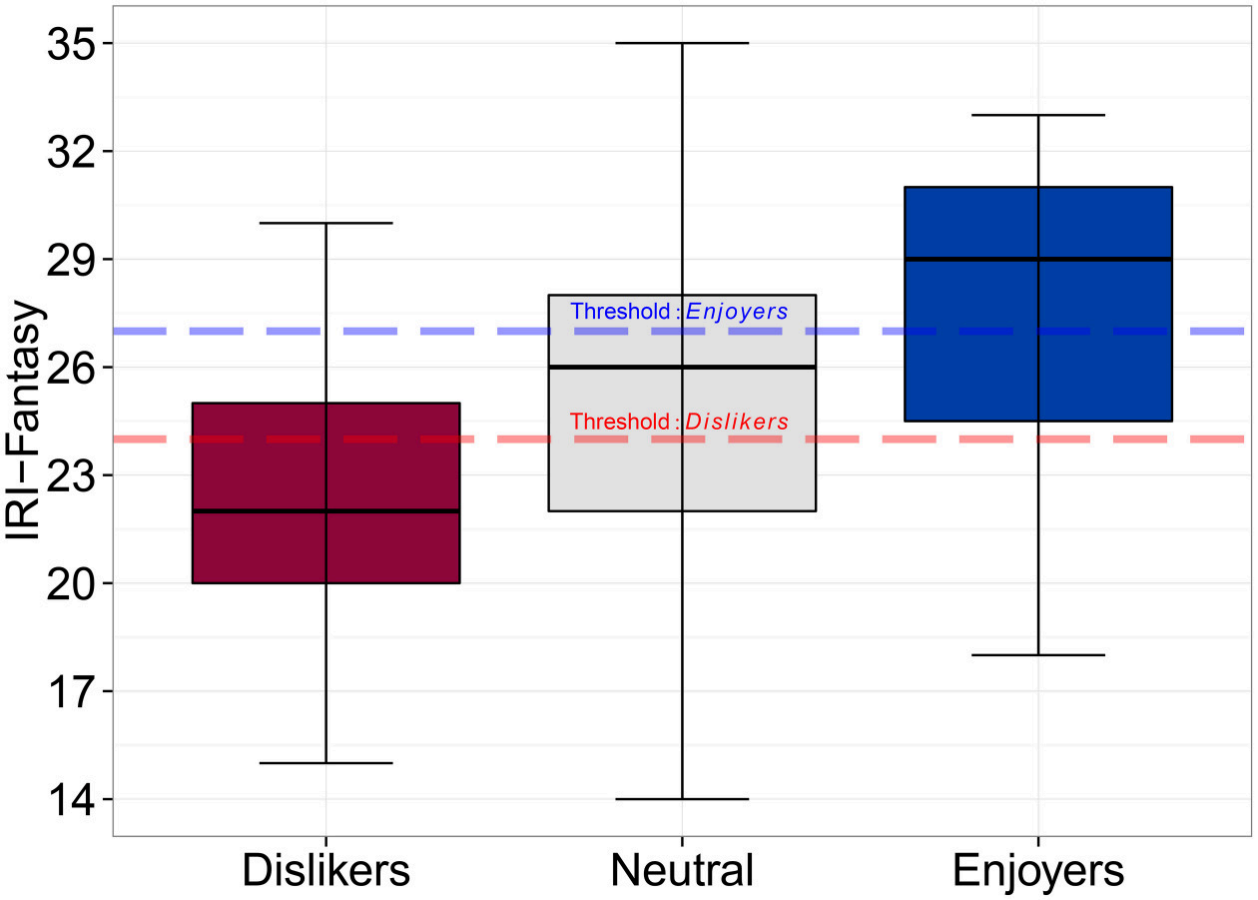
**Subscale of empathy (***IRI-Fantasy***) scores for all participants across the lower (Dislikers) and upper (Enjoyers) quintiles of ***Moving sadness*****. The dashed lines show the optimal cutpoints for both extremes based on the ROC analysis.

The optimal cut-off points, determined by simultaneously maximizing sensitivity and specificity (López-Ratón et al., 2014), offer plausible thresholds for diagnosing people as sadness enjoyers. Since there were only marginal differences between the best classification predictors, variable parsimony is the best way of determining the most discriminating feature. This suggests that *IRI-Fantasy* is the most efficient feature for this purpose; it has similar success rate to *ECS* and *IRI-Empathy*, but it only consists of seven items, whereas the other predictors have over two (15 in *ECS*) to four (28 in *IRI-Empathy*) times the number of items. Figure 2 illustrates the relationship between *IRI-Fantasy* and *Moving Sadness*.

For the purposes of comparison, the top quintiles in the two other factors representing the emotions experienced in response to sad music were explored. Those in the highest quintile in *Nervous sadness* could also be predicted with *IRI-Empathy* (AUC = 64.2, CI_95_ = 51.0–77.4) to a moderate degree, but not from any other variables (AUCs < 0.60). The extreme quintiles of *Relaxing sadness* could not, however, be reliable distinguished by any of the variables (all AUCs < 0.60).

## 4. Discussion

This study has shown that unfamiliar, instrumental sad music can evoke strong emotional responses in listeners. These responses can be characterized in terms of three underlying factors: *Relaxing sadness, Moving sadness*, and *Nervous sadness*. *Relaxing sadness* was characterized by felt and perceived peacefulness and positive valence, while *Nervous sadness* was related to felt anxiety, perceived scariness, and negative valence. *Moving sadness*, the factor that became the central focus of this manuscript, captured an intense experience that involved feelings of sadness and being moved, liking, and perceived sadness. Experiences of *Relaxing sadness* and *Moving sadness* received comparably high mean ratings, whereas the factor related to negative emotional experiences, *Nervous sadness*, received very low mean ratings. It may be that these kinds of strongly negative experiences that are sometimes evoked by sad music are intrinsically linked with personal memories (Van den Tol and Edwards, 2013; Peltola and Eerola, 2016), and thus seldom experienced in the context of unfamiliar sad music. The three-factor structure obtained in this study is consistent with recent studies (Peltola and Eerola, 2016), despite small differences in the labeling and interpretation of the different factors.

To complement and corroborate the self-reports of felt emotions, we also measured physiological indices of felt emotions (electrodermal activity and HRV), as well as sadness-related judgment biases in emotional evaluations. The physiological indices—the spectral HRV components in particular—provided evidence of increased sympathetic activity during the music listening compared to baseline. This implies that listening to the unfamiliar sad music did not merely evoke relaxation, but lead to increased emotional arousal. The indirect facial expression judgment task confirmed that those who reported experiencing more *Nervous sadness* were indeed experiencing a more negative emotional state, as they exhibited diminished happiness evaluations. However, this was not the case for those who experienced more *Moving sadness*. This differing pattern of judgment biases suggests that while those with high scores of *Moving sadness* may have indeed experienced a sad emotional state, this experience was not negative (unlike in the case of those with high scores of *Nervous sadness*). *Relaxing sadness* was not significantly associated with any judgment biases.

Experiences of *Moving sadness* were predicted by trait empathy (the Fantasy subscale in particular) and sensitivity to emotional contagion. The Fantasy subscale has previously been associated with preference for sad-sounding music (Garrido and Schubert, 2011a; Vuoskoski and Eerola, 2011; Vuoskoski et al., 2012; Kawakami and Katahira, 2015), and—in line with these findings—liking ratings also contributed to *Moving sadness* in the present study. Furthermore, the present study demonstrated that Fantasy could be used as an efficient diagnostic predictor to identify those participants who scored in the highest quintile in *Moving sadness*—a group labeled as “Sadness enjoyers.” The other types of experiences evoked by sad music—*Relaxing sadness* and *Nervous sadness*—were not strongly associated with most of the background variables, but both correlated (positively and negatively, respectively) with subjective evaluations of *Quality of life*.

The fact that Fantasy and Emotional contagion best predicted experiences of *Moving sadness* suggests that the tendency to engage in narrative transportation (i.e., the process of identifying with fictional characters and “losing” oneself in the story (Green and Brock, 2000; Hall and Bracken, 2011), as well as the tendency to “catch” the emotions of others contribute to the experience of *Moving sadness*. As noted earlier, *Moving sadness* captured both the experience of intense emotions and the enjoyment of sad music. Although Fantasy is typically considered to be a subscale of cognitive rather than affective empathy (along with Perspective-taking), previous work has associated it with the experience of more intense emotions in response to films (Harris et al., 2000) and music (Vuoskoski and Eerola, 2012). Furthermore, it has been shown that perspective-taking actually facilitates the experience of affective empathy (Batson et al., 2003). Although it is perhaps unsurprising that those who have a tendency to engage in immersive perspective-taking in fictional contexts also experience more intense emotions in response to sad music (and enjoy it more), it is less clear why Emotional contagion contributed to experiences of *Moving sadness* rather than to experiences of the more negative *Nervous sadness*.

A possible explanation may be offered by the involvement of *Empathic concern* (sometimes referred to as sympathy, Eisenberg et al., 1994), the other empathy-subscale that was positively correlated with experiences of *Moving sadness*. Eisenberg and colleagues (Eisenberg et al., 1994; Decety and Jackson, 2004) have proposed that an individual's self-regulation abilities determine whether an empathic response leads to experiences of Empathic concern or Empathic distress. While both *Empathic concern* and *Personal distress* are forms of affective empathy, *Empathic concern* (or sympathy) is associated with other-focused, pro-social behavior, whereas *Personal distress* is an aversive, self-focused response involving feelings of discomfort and anxiety (Davis, 1983; Eisenberg et al., 1994). Consequently, one might have expected that *Personal distress* would have predicted experiences of *Nervous sadness*, as this factor was related to negative and aversive emotional experiences. Although no statistically significant correlations emerged between *Nervous sadness* and the trait empathy subscales, a non-significant trend between *Personal distress* and *Nervous sadness* was observed (*r* = 0.18, *p* = 0.075). It may be that the low mean ratings of *Nervous sadness* (i.e., the lack of aversive, anxious responses to the sad musical stimulus) prevented any statistically significant correlations from emerging.

Although a number of previous studies have investigated the contribution of certain background variables to liking for sad music (Garrido and Schubert, 2011a; Vuoskoski et al., 2012; Kawakami and Katahira, 2015) and the emotional responses induced by it (Vuoskoski and Eerola, 2012; Vuoskoski et al., 2012), the present study has explored a much more exhaustive set of background variables than any of the previous studies. It is worth noting that many of the background variables reported in previous studies attempting to explain individual differences in responses to sad music—such as *Nostalgia proneness* and *Absorption*—did not significantly predict any of the emotion factors in the present study. Nostalgia has often been mentioned as one of the variables explaining the enjoyment of music-induced sadness (Barrett et al., 2010; Taruffi and Koelsch, 2014), but it may be that experiences of nostalgia are more often evoked by familiar (rather than unfamiliar) sad music. *Absorption* has also been previously associated with liking for sad music (Garrido and Schubert, 2011b; Hogue et al., 2016) and the intensity of emotional responses evoked by it (Kreutz et al., 2008), but it did not predict emotional responses in the present study. It may be that some of the differences observed between the results of previous studies and the present study are due to methodological differences, as many of the previous studies (Garrido and Schubert, 2011a, 2013; Taruffi and Koelsch, 2014; Hogue et al., 2016) have employed survey measures without actual music listening. Survey studies cannot differentiate between responses to familiar and unfamiliar sad music, and thus the links observed between background variables and responses to sad music may be attributable to sources external to music.

The decision to focus on unfamiliar music was made to control for external explanations and influences (Eerola and Vuoskoski, 2012), but this focus limited the choice and number of stimuli. Having only a single musical excerpt as the experimental stimulus may have caused untoward emphasis on the particular example. However, prior studies with this particular music excerpt confer additional information about its effectiveness in inducing sadness in listeners, and the kinds of associations and emotion induction mechanisms that are commonly reported by listeners (Vuoskoski and Eerola, 2012, 2015). A recent investigation utilizing sad-sounding music examples from different genres (including the excerpt used in the present study) has shown that the structure of emotional responses evoked by sad music is consistent across different genres (Vuoskoski and Eerola, submitted).

Listening experiments may not be able to address the functional uses of listening to sad music, which are often related to certain states of mind or situations (Juslin et al., 2011; DeMarco et al., 2015). Regulatory uses of sad music are important for listeners' enjoyment, and it has been documented that listeners in a sad affective state tend to prefer mood-congruent music (Friedman et al., 2012) although conflicting results have also been reported (Hunter et al., 2011; DeMarco et al., 2015). Nonetheless, such uses are driven by external causes, and therefore do not hold the key for understanding the appeal of sad music outside this context.

The sample size of the present study was moderately small, albeit larger than in most experiments on sad music (Juslin et al., 2013; Kawakami and Katahira, 2015). More importantly, unlike all previous studies investigating responses to sad music, the participants of the present study were drawn from a representative sample. This allows us to interpret the results not only as the preferences and habits of those who love music—as in past survey studies utilizing convenience samples (Van den Tol and Edwards, 2013; Taruffi and Koelsch, 2014), but as a more general trend applicable to the general population. This is a crucial distinction in determining which, if any, background variables are associated with the observed inter-individual differences, since convenience samples may have non-representative personality profiles (Rawlings et al., 2000; Delsing et al., 2008).

Gender can be seen as another caveat in the interpretation of the results. The experiences of *Moving sadness* exhibited significant gender differences, and the diagnostic prediction of Enjoyers and Dislikers (with trait empathy) was satisfactory for women only. There are at least two issues potentially contributing to this gender asymmetry. Firstly, being moved by fiction seems to be rarer, or at least less intensive in men (Oliver et al., 2000; Oliver, 2007; Menninghaus et al., 2015). This may be partly due to culturally acquired biases, where men are not suppose to show emotional responsiveness to the same degree as women, thus producing notable differences in self-report measures but less divergence in behavioral measures (Brody and Hall, 2008). Secondly, the facets of empathy seem to differ subtly between men and women; women score higher in emotion recognition and social sensitivity, and tend to recruit mirror neurons areas more strongly in tasks related to emotional contagion (Schulte-Rüther et al., 2008; Derntl et al., 2010).

The results of the present study offer several theoretical implications. First, the results suggest that external factors such as lyrics, memories, or familiarity are not crucial for the enjoyment of sad music (Vuoskoski et al., 2012; Tahlier et al., 2013; Taruffi and Koelsch, 2014; Brattico et al., 2016). We minimized the contribution of these external factors by exposing listeners to unfamiliar sad music, but this did not eliminate the positive, enjoyable, and moving experiences evoked by the music. How this emotional experience is constructed is something that cannot yet be fully answered, yet certain strands of explanation appear more plausible than others. Numerous explanations for the paradox of enjoyable sadness in fictional contexts have been offered, although most of them are specific to films and other narratives, and involve higher-level cognitive reappraisals (Oliver, 1993; De Wied et al., 1995; Ahn et al., 2015).

Although fictionally evoked sadness does not have the negative real-world implications associated with real-life sadness, it can feel genuine and involve similar psychological, physiological, and biochemical reactions due to embodied simulation of the emotion (Niedenthal, 2007). The association between trait empathy and *Moving sadness* observed in the present study is congruent with this notion, demonstrating how those who have a higher capacity for engaging with the experiences of others also do so in the context of music listening, and also seem to enjoy the experience more. But, as the factor loadings of *Moving sadness* illustrate, the empathic emotion experienced in response to sad music is not simply sadness, but a mixture of being moved, sadness, and enjoyment. This transformation from the perception of sadness to enjoyable experiences of being moved requires a consideration of how the lack of real-world repercussions actually lead to the experience of strong, enjoyable emotions rather than just neutral or weakly negative emotions.

In a real-world experience of loss, the function of sadness is to show appropriate social signals and activate a defensive reaction comprising specific endocrine responses, especially when crying is involved (Gračanin et al., 2014). The biochemical response to loss consists of a general stress response (involving catecholamines, the HPA axis, corticotrophin-releasing hormone and cortisol (Uvnäs-Moberg et al., 2014) as well as an attachment-specific stress response that involves the dopamine and oxytocin systems that help the mind to cope with a loss (Depue and Morrone-Strupinsky, 2005). Oxytocin in particular is known to increase calmness and a general sense of well-being (Heinrichs et al., 2003), and is assumed to be crucial in self-soothing and social engagement (Uvnäs-Moberg et al., 2014). When engaging with fictional sadness, the individual effectively simulates a real experience of sadness, and the following hormonal and physiological responses only differ from real loss in terms of intensity. When this cocktail of functional endocrine response is released without a real-world loss, it is hypothesized to lead to experiences of being moved and comforted, and in other words, to pleasure (Huron, 2011).

Explanations pertaining to higher-level processes consider pleasure derived from fictionally evoked sadness as a result of cognitive appraisal. According to one account (Ryff, 1989), witnessing fictional sadness (loss, tragedy) allows the viewer to gain perspective to his or her own life, and such reassessment is satisfying. Empirical support for this view has been obtained in the context of tragic films (De Wied et al., 1995). However, this explanation might be more relevant for fictional narratives rather than for something as abstract as music, although it has been argued that listeners can also “hear” music as a narrative or as the emotional experiences of a virtual person (Cohen, 2001; Levinson, 2006). Another account suggests that aesthetic experiences are subject to a set of rules that revolve around the concept of beauty. Furthermore, such experiences are inherently enjoyable since they happen in the fictional domain, and are thus without any real-world consequences (Kivy, 1990; Robinson, 1994). More specifically, Juslin (2013) proposes that music-induced sadness is enjoyable only when it happens together with a percept of beauty, and that this enjoyment is due to aesthetic appreciation—not the induced emotion. Although parts of this explanation are compelling, we argue that it actually puts off the real, underlying explanation, since “beauty” is just another way of describing an aesthetic experience that is moving, memorable, and enjoyable.

The present study advanced the understanding of the paradoxical enjoyment of music-induced sadness by discovering the main kinds of emotions experienced and by minimizing the external causes of emotions. Most importantly, the strong, enjoyable responses to sad music were associated with empathy-related traits to such degree that it was possible to diagnostically identify those who derive pleasure from sad music from those who do not. A follow-up study using a similar set of constraints and both responsive and unresponsive participants could address how the simulation of a negative emotion such as sadness could lead to enjoyment in a fictional context. Another vital piece of this jigsaw would be to understand and track the momentary evolution of the emotional experience, since some form of transformation—from a simulated negative emotion to an experienced positive emotion—is taking place. Other forms of fiction, such as films, for instance, have already provided insights into the paradoxical pleasure evoked by fictional tragedy (De Wied et al., 1995; Oliver et al., 2000), but whether these forms of art would also be able to create a transformation of emotional experience without the high-level narrative devices, is an open question. The results of the present study—eliminating most extramusical causes of emotions—suggest that music may have a rather direct route to our emotions. To quote Oliver Sacks, “Music can pierce the heart directly; it needs no mediation. One does not have to know anything about Dido and Aeneas to be moved by her lament for him; anyone who has ever lost someone knows what Dido is expressing” (Sacks, 2007, p. 301). While we currently do not know all the details of the transformations involved in this process, identifying the correct pieces of puzzle is the first step in unraveling the perennial paradox of fiction and creating better-informed practices of arts-based therapy and rehabilitation.

## Author contributions

TE conceived the experiment, TE and JV conducted the experiment, TE, JV, and HK analyzed the results. All authors reviewed the manuscript.

## Funding

This work was financially supported by the Academy of Finland Grant 270220 (Surun Suloisuus).

### Conflict of interest statement

The authors declare that the research was conducted in the absence of any commercial or financial relationships that could be construed as a potential conflict of interest.
